# Growth of Infants Fed Formula with Evolving Nutrition Composition: A Single-Arm Non-Inferiority Study

**DOI:** 10.3390/nu9030219

**Published:** 2017-03-01

**Authors:** Johannes Spalinger, Andreas Nydegger, Dominique Belli, Raoul I. Furlano, Jian Yan, Jerome Tanguy, Sophie Pecquet, Frédéric Destaillats, Delphine Egli, Philippe Steenhout

**Affiliations:** 1 Department of Gastroenterology and Hepatology, Children’s Hospital, CH-6000 Lucerne, Switzerland; 2 Department of Pediatric Gastroenterology, University Children’s Hospital, CH-1011 Lausanne, Switzerland; Andreas.Nydegger@chuv.ch; 3 Department of Pediatrics, University Hospital of Geneva, CH-1205 Geneva, Switzerland; Dominique.Belli@hcuge.ch; 4 Department of Pediatrics, University Children’s Hospital Basel (UKBB), CH-4031 Basel, Switzerland; raoul.furlano@ukbb.ch; 5 Nestlé Nutrition Research, King of Prussia, PA 19406, USA; jian.yan@rd.nestle.com; 6 Nestlé Research Center, CH-1000 Lausanne, Switzerland; jerome.tanguy@rdls.nestle.com; 7 Nestlé Nutrition Research, CH-1800 Vevey, Switzerland; sophie.pecquet@nestle.com; 8 Nestlé Product Technology Center, CH-1800 Vevey, Switzerland; frederic.destaillats@nestle.com; 9 Nestlé Nutrition Medical, Scientific and Regulatory affairs Unit, CH-1800 Vevey, Switzerland; delphine.egli@nestle.com; 10 Nestlé Health Science, CH-1800 Vevey, Switzerland; psteenhout@me.com

**Keywords:** human milk, evolving nutritional composition, infant formula, protein, staged-formula delivery system, WHO growth standard, personalized nutrition

## Abstract

The nutritional composition of human milk evolves over the course of lactation, to match the changing needs of infants. This single-arm, non-inferiority study evaluated growth against the WHO standards in the first year of life, in infants consecutively fed four age-based formulas with compositions tailored to infants’ nutritional needs during the 1st, 2nd, 3rd–6th, and 7th–12th months of age. Healthy full-term formula-fed infants (*n* = 32) were enrolled at ≤14 days of age and exclusively fed study formulas from enrollment, to the age of four months. Powdered study formulas were provided in single-serving capsules that were reconstituted using a dedicated automated preparation system, to ensure precise, hygienic preparation. The primary outcome was the weight-for-age *z*-score (WAZ) at the age of four months (vs. non-inferiority margin of −0.5 SD). Mean (95% CI) *z*-scores for the WAZ (0.12 (−0.15, 0.39)), as well as for the length-for-age (0.05 (−0.19, 0.30)), weight-for-length (0.16 (−0.16, 0.48)), BMI-for-age (0.11 (−0.20, 0.43)), and head circumference-for-age (0.41 (0.16, 0.65)) at the age of four months, were non-inferior. Throughout the study, anthropometric *z*-scores tracked closely against the WHO standards (within ±1 SD). In sum, a four-stage, age-based infant formula system with nutritional compositions tailored to infants’ evolving needs, supports healthy growth consistent with WHO standards, for the first year of life.

## 1. Introduction

Human milk is the gold standard for infant nutrition and provides all nutrients to support normal growth during the first six months of life [1]. However, if the mother cannot or chooses not to breastfeed, appropriate alternatives must be available. Infant formulas have been developed, based on the composition of breast milk and successfully meet the nutritional needs of healthy infants, while striving to match the associated health benefits of breastfeeding [2]. However, some differences are still observed between breast-fed and formula-fed infants, such as the kinetics of early growth, which may be associated with the risks of obesity and chronic disease in later life [3,4]. 

One reason for the differences between breast-fed and formula-fed infants may be the different nutrient composition of human milk, compared with infant formulas. Human milk is a dynamic fluid, changing substantially in composition, especially during the first four to six months of lactation [2,5]. In contrast, formula composition is relatively static and must meet all of an infant’s nutritional requirements. Changes in protein concentration over the course of lactation illustrate the dynamic nature of human milk composition. Protein levels are relatively high in colostrum and fall significantly during the first weeks of lactation. A systematic review of human milk protein and amino acid composition [6] reported a median protein content of 2.06 g/100 mL in colostrum collected 0–5 days after delivery, and 1.57 g/100 mL in milk collected 16–30 days after delivery; the median protein content further decreased to 1.10 g/100 mL in mature milk collected 90–360 days after delivery. The protein content of infant formula is higher than that of mature human milk for two reasons: firstly, the essential amino acid content of the proteins in infant formula differs from that of human milk and higher protein levels must be present to provide all of the essential amino acids in adequate quantities [7,8]; secondly, the amino acid requirements are higher for infants during the first two months of life than at later ages, and formula must meet these requirements [9]. In addition to protein levels, energy levels also differ between infant formula and human milk. Recent studies [2] suggest that the caloric density of human milk is close to 550 kcal/L, which is lower than that of infant formulas (generally 670 kcal/L), while the energy requirements per kg of body weight fall substantially during the first few months of life (~23% decrease from one to six months of age) [10]. Therefore, formula-fed infants may receive more calories than they need [11]. For these reasons, the development of age-based infant formulas that are tailored to more accurately meet the nutritional requirements of infants is desirable, despite many difficulties in designing a formula that provides similar levels of bioavailable energy and protein as human milk.

Powdered infant formula is primarily available in multi-serve packaging, which requires the measurement of the product with specific scoops and multiple manual preparation steps. Preparing the correct concentration of formula is imperative, in order to provide appropriate amounts of nutrients. Unfortunately, parents sometimes make mistakes during the preparation of formula [12,13]. Incorrectly reconstituted formulas may provide calories, protein, and other nutrients, either in excess of the infant’s nutritional requirements, or as insufficient for meeting the nutritional needs for normal growth and development. Thus, a reliable single-serving reconstitution system has been developed and was employed for formula reconstitution in the present study. 

We tested the hypothesis that an advanced nutrition system, comprised of a series of four age-based infant formulas with a gradually reduced protein content, would provide adequate nutrition to support healthy infant growth. This nutrition system provides formula capsules in four stages, based on infant age (1, 2, 3–6, and 7–12 months), that are designed to more closely match the nutrient content in human milk over the course of lactation. The primary objective of this single-arm, non-inferiority study was to determine whether growth between 0–4 months of age in infants exclusively fed with the study formulas, was comparable with the WHO 2006 Child Growth Standards [14].

## 2. Materials and Methods

This study was a prospective, open-label, multi-center, single-arm, 12-month study. Formula-fed infants were enrolled at ≤14 days of age from four hospitals in Switzerland: Children’s Hospital, Geneva; University Children’s Hospital Basel (UKBB), Basel; Children’s Hospital, Lucerne; and Center University Hospital Vaudois (CHUV), Lausanne. The study was conducted between April 2012 and July 2014, in compliance with the Declaration of Helsinki and the International Conference on Harmonization guidelines for Good Clinical Practice, and was approved by the Independent Ethics Committee at each participating institution. Signed written informed consent was obtained from the parent or legal guardian of each subject, prior to enrollment.

### 2.1. Subjects

Healthy term infants ≤14 days of age, whose mothers had a BMI between 18.5–30 kg/m^2^ at the start of pregnancy and had previously decided to feed their child exclusively with formula, were invited to participate. Additional inclusion criteria included: (i) full-term newborn (≥37 weeks gestation); (ii) birth weight ≥2500 g and ≤4500 g; (iii) infants from birth to 14 days of age at the time of enrollment; and (iv) newborn’s mother voluntarily elected to exclusively formula-feed her newborn. Infants were excluded if they had a congenital illness or malformation that could affect normal growth (especially immunodeficiency); had a mother who had an abnormal pre-pregnancy BMI (<18.5 or ≥30 kg/m^2^), type 1 or type 2 diabetes, or a chronic infectious disease; had parents/caregivers who could not be expected to comply with treatment; or were currently participating in another nutritional interventional clinical trial (except for vaccination studies). 

### 2.2. Study Product

Four infant formulas with age-specific nutritional compositions were tested in the study. These formulas were consumed consecutively, with the 1st formula for the first month of age, 2nd formula for the second month of age, 3rd formula for 3–6 months of age, and 4th formula for 7–12 months of age (BabyNes™, Nestlé, Switzerland). These formulas provided all of the essential nutrients and had protein concentrations that gradually decreased from the 1st to 4th stage, from 1.51 to 1.31 to 1.18 to 1.17 g/100 mL (Table 1), which is consistent with the decreasing protein content in human milk over the course of lactation. All of the formulas contained intact cow’s milk protein and the 1st to 3rd formulas were whey protein dominant (whey:casein ratio of 70:30; whey:casein ratio of 50:50 in the 4th formula). The formulas also contained the probiotic *Bifidobacterium lactis CNCM* I-3446, at the level of 1 × 10^6^ CFU/g. Formula powders were provided in single-serving capsules along with an automated formula preparation system, which delivered precisely reconstituted hygienic formula. Single-serving capsules were offered in two sizes for the 1st to 3rd formulas, which allowed reconstitutions of 90 or 120 mL of the 1st formula, 150 or 180 mL of the 2nd formula, and 180 or 210 mL of the 3rd formula. One capsule size for the 4th formula was available, which allowed a reconstitution of 240 mL. Infants consumed age-appropriate formulas ad libitum, and parents were advised to select the size of age-appropriate formulas, according to the infant’s appetite. More than one capsule could be used at one feeding occasion and parents were advised to discard the leftover if infants did not consume all of the prepared formula. Infants were exclusively fed the study formulas from enrollment to four months of age; complementary foods were allowed from four months of age, until the study ended at 12 months of age.

### 2.3. Study Visits and Outcome Measures

The study consisted of a baseline visit at enrollment, followed by visits at 0.5, 1, 2, 4, 6, 9, and 12 months of age. At the baseline visit, demographic information was collected and anthropometrics (infant weight, length, and head circumference) were obtained. Anthropometrics at the time of birth were also recorded. At each subsequent visit, anthropometric data were obtained and formula intake was also collected via a diary, in which parents were also instructed to record information on illness symptoms and episodes, and concomitant treatments. This latter information was used for the assessment of adverse events (AEs).

The primary study outcome was the weight-for-age *z*-score (WAZ) at the age of four months, compared with the WHO 2006 Child Growth Standard [14]. Secondary outcomes included the WAZ at other time points, as well as additional anthropometric measures compared with the WHO standards [*z*-scores of length-for age (LAZ), weight-for-length (WLZ), BMI-for-age (BMIAZ), and head circumference-for age (HCAZ)], formula intake, and reported AEs throughout the study. 

Anthropometrics were measured by trained personnel, following standard procedures. Briefly, weight was measured to the nearest 10 g. Infants were weighed without clothing or a nappy/diaper on electronic weighing scales and the same scales were used for all infants during all visits. For recumbent length measurements, infants were measured using a standardized length board. At least two people were present to maintain proper body alignment and full body extension with flexed feet. Head circumference measurements were obtained using a standard non-elastic, plastic-coated measuring tape. The measurement was taken approximately 2.5 cm above the eyebrows, directly over the largest circumference of the skull. WAZ, LAZ, WLZ, BMIAZ, and HCAZ were then calculated, based on the WHO 2006 Child Growth Standards using a SAS Macro [15].

Parents were instructed to record information about formula intake in a diary, provided during the baseline visit. Intake was recorded on the first three days and last three days that infants were fed each study formula. Parents also recorded the formula intake for the three days prior to and three days following the study visits that corresponded with no change in study formula (i.e., at age four months and age nine months). 

Parents were also instructed to record information on illness symptoms and episodes, as well as concomitant treatments in the diary. This information was then reviewed by the study investigators or nurses at each visit, before being recorded in the study database as AEs. AEs were then coded and categorized by a single physician who was not involved in the conduct of the study, using the Medical Dictionary for Regulatory Activities (MedDRA; version 14.1, International Federation of Pharmaceutical Manufacturers and Associations, Geneva, Switzerland) System, Organ, and Class (SOC) categories, as well as Preferred Terms (PTs). 

In light of existing data linking excessive weight gain in infancy to the risk of obesity and related morbidities in later life [16,17], we conducted a post hoc analysis to explore weight gain patterns, based on the change in WAZ during the period of exclusive formula feeding from birth to four months of age (WAZ at four months minus WAZ at birth), as well as during the entire study period (WAZ at 12 months minus WAZ at birth). The change in WAZ was classified as “slow” (<−0.67 SD), “gradual” (−0.67 SD to 0.67 SD), or “rapid” (>0.67 SD), as previously described [18], which are considered equivalent to “downward”, “average”, and “upward” crossings of major weight percentiles in a growth chart (i.e., WHO 2006 Child Growth Standards).

### 2.4. Statistical Analysis

The initial sample size calculation determined that, in order to show a mean WAZ significantly greater than the non-inferiority margin of −0.5 SD (primary outcome), with a standard deviation of 1.2 SD at the α level of 0.025 with 90% power in a 1-sided *t*-test, 63 infants were needed. The non-inferiority margin of −0.5 SD, which is equivalent to a 3 g/day weight gain difference from 0–4 months of age, was considered clinically relevant, as per American Academy of Pediatrics recommendations [19]. With an estimated drop-out rate of approximately 20%, an enrollment target of 80 infants was planned. However, slow subject recruitment limited the ability to achieve the originally planned sample size and the enrollment was stopped after a total of 33 infants were enrolled. 

Anthropometric *z*-scores were analyzed using a linear multivariate regression model, accounting for repeated measurement. Structural components of the model defined by the fixed effects included birth z-scores, infant sex, study center, visits, smoking status of the mother (if significant at 0.1 level), and visits × infant sex. The correlation between repeated measurements was described with an unstructured variance-covariance matrix, as a first attempt. In the case of non-convergence, an alternative matrix was selected, according to Akaike criteria. Estimated means and 95% CIs of z-scores at each visit were calculated from the model using weighted estimates, correcting for any covariate imbalance. 

A confidence interval approach was used to assess the non-inferiority of the primary outcome, compared with the WHO standard. The estimated WAZ mean and 95% CI at the age of four months was compared to the non-inferiority margin of −0.5 SD; non-inferiority was demonstrated if the lower limit of the 95% CI was above −0.5 SD. The confidence interval approach was also used to assess the non-inferiority of secondary anthropometric outcomes, compared with the WHO standards. 

For additional outcomes in the single-arm study, including formula intake, morbidities, and weight gain patterns based on the categories for WAZ changes, descriptive statistics are presented. 

Due to a limited amount of missing data, a sensitivity analysis based on the missingness mechanism was judged nonrelevant. All analyses were conducted in both the full analysis set (FAS), which was defined as all enrolled infants who had at least one serving of the test infant formula, and the per protocol (PP) set, which was defined as the set of compliant subjects. Non-compliance included (i) violating inclusion/exclusion criteria; (ii) changing regimen due to a life-threatening event; and (iii) consuming complementary foods before four months of age, defined as taking four or more teaspoons (i.e., 20 g) of complementary foods per day. The PP set is considered the appropriate analysis population for non-inferiority testing [20,21], and therefore, the PP results are reported here. Results for the primary outcome (WAZ at the age of four months) are reported from both the PP and FAS sets.

All analyses were conducted using the software package SAS version 9.3 (SAS Institute Inc., Cary, NC, USA). The data presented are estimated means (95% CIs), unless otherwise noted. 

## 3. Results

### 3.1. Study Population

**Figure 1:**
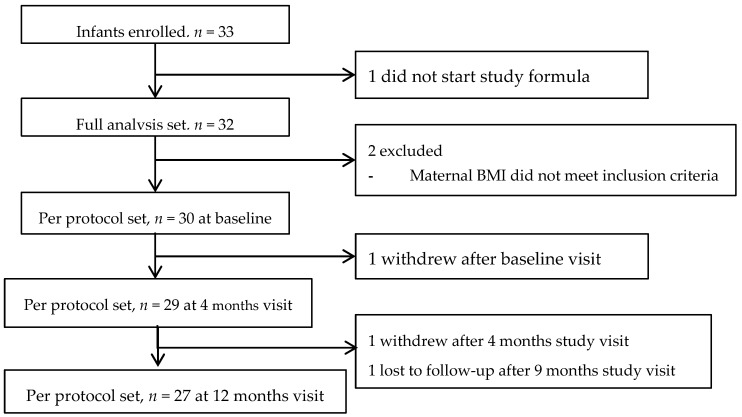
Subject disposition.

The disposition of the study subjects is shown in Figure 1. A total of 33 infants were enrolled. One subject dropped out before starting feedings with the study formula, thus the FAS included 32 infants. The PP set included 30 subjects because the mothers of two of the 32 infants did not meet the inclusion criteria for BMI, and as a result, they were excluded from the PP set. The median (interquartile range, IQR) follow-up duration, defined as the time between the date of the first study formula intake, up to the date of the last study visit, of the PP set was ~11.7 (11.6–11.8) months. The median (IQR) compliance rate (defined as the days on which formula was consumed divided by the total follow up duration × 100%) was 100% (99.4%–100%). 

Demographic and baseline characteristics for the PP set are shown in Table 2. Briefly, both sexes were equally represented and more than half were delivered via Caesarean section. On average, infants were enrolled in the study when they were 8.7 (SD 4.2) days old and they started consuming the 1st study formula at the age of 9.2 days (SD 3.9). Mothers had a mean (SD) age of 35 (4.7) years at enrollment and had a mean BMI of 23.30 (SD 2.57) kg/m^2^ prior to pregnancy. 

### 3.2. WAZ

**Figure 2:**
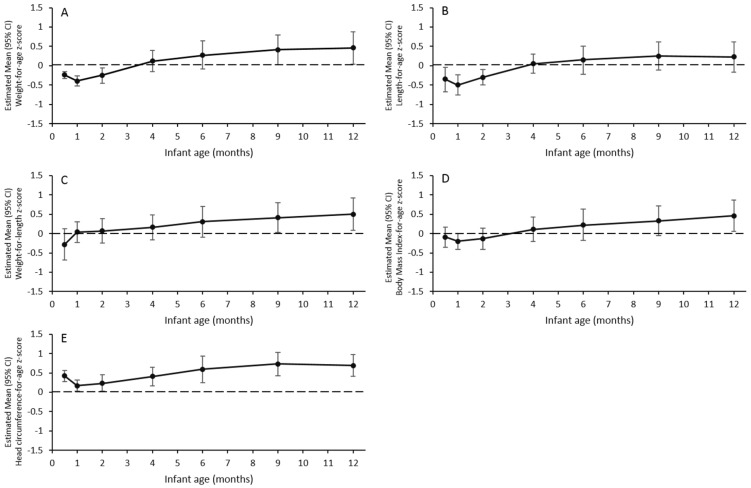
Anthropometric *z*-scores compared to the WHO 2006 Child Growth Standards. Data presented are estimated means (95% CIs) of *z*-scores for weight-for-age (**A**), length-for-age (**B**), weight-for-length (**C**), body mass index-for-age (**D**), and head circumference-for age (**E**) calculated from linear repeated mixed models. Fixed effects included corresponding birth *z*-scores, infant sex, study center, visits, smoking status of the mother (if significant at 0.1 level), and visits × infant sex. *n* = 17, 29, 29, 29, 28, 28, and 27, at age 0.5, 1, 2, 4, 6, 9, and 12 months, respectively.

In the PP set, the estimated mean (95% CI) WAZ at four months was 0.12 (−0.15, 0.39) and the lower limit of the 95% CI was above the −0.5 SD non-inferiority margin (Figure 2A). The results in the FAS were consistent with this finding: the estimated mean (95% CI) for WAZ in the FAS was 0.07 (−0.18, 0.33) and the lower limit of the 95% CI was above the −0.5 SD non-inferiority margin. The mean WAZ increased throughout the study (Figure 2A). For the entire 12-month study period, the upper limits of the 95% CIs for WAZ were <1 SD, while the lower limits of the 95% CIs were >−0.5 SD.

### 3.3. LAZ, WLZ, BMIAZ, and HCAZ

The results for other anthropometric *z*-scores at four months of age were similar to that of WAZ. The lower limits of the 95% CIs for LAZ (Figure 2B), WLZ (Figure 2C), BMIAZ (Figure 2D), and HCAZ (Figure 2E) at the age of four months, were all above the −0.5 SD non-inferiority margin. The mean LAZ was significantly <0 at ages 0.5, 1, and 2 months (similar to that of WAZ), but did not differ from 0 at later time points. The mean WLZ did not differ from 0 from the baseline, through to an age of six months, but was significantly >0 at nine and 12 months. BMIAZ did not differ from 0 at any time point, except at 12 months, when it was significantly >0. HCAZ was significantly >0 at all time points; this particular finding is consistent with recent observations [18,22], which showed that WHO head circumference data are consistently lower than measurements from large studies of economically advantaged children. Collectively, these findings suggest that further considerations are needed in regard to using a single international head circumference standard. For the entire 12-month study period, the upper and lower limits of the 95% CIs for these anthropometric *z*-scores were within ±1 SD.

### 3.4. Weight Gain Pattern Based on Change in WAZ

**Figure 3:**
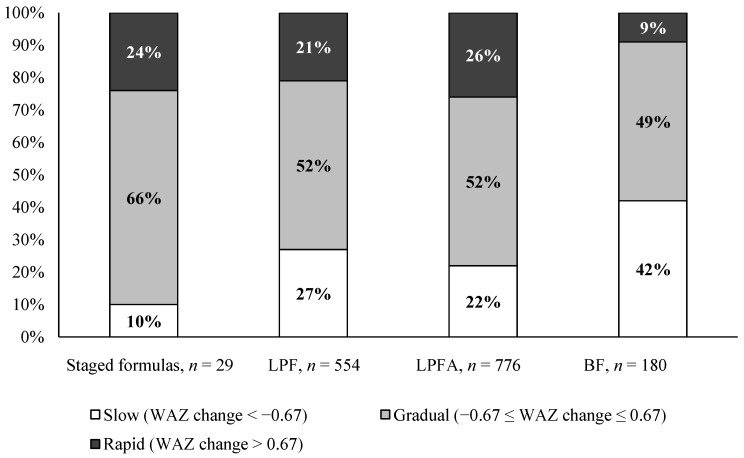
Weight gain pattern between birth and four months of age.Data are presented as the percentage of infants in weight gain categories based on WAZ change as slow (<−0.67), gradual (−0.67 to 0.67), or rapid (>0.67), for infants fed staged formulas in the current study, and infants included in a pooled analysis [18] who were fed lower protein formula (LPF, 1.8 g protein/100 kcal) or lower protein formula with active ingredients (LPFA, 1.8 g protein/100 kcal with prebiotics, probiotics, or both), or breastfed infants (BF). WAZ change was calculated using WAZ at four months of age, minus WAZ at birth. Graphs for LPF, LPFA, and BF were created, based on the data published in Alexander et al. [18], after obtaining the journal’s permission.

From birth to four months of age, the mean change (SD) in WAZ was 0.11 (0.8); the proportion of infants in “slow”, “gradual”, and “rapid” categories was 10%, 66%, and 24%, respectively (Figure 3). For growth from birth to 12 months of age, the mean change (SD) in WAZ was 0.49 (1.2); the proportion of infants in “slow”, “gradual”, and “rapid” categories was 22%, 30%, and 48%, respectively. In a recent large-scale pooled analysis of 11 randomized controlled trials [18], the proportion of infants with a WAZ change from birth to four months of age in “slow”, “gradual”, and “rapid” categories, was 27%, 52%, and 21% among infants fed lower protein formulas without active ingredients (1.8 g protein/100 kcal); was 22%, 52%, and 26% among infants fed lower protein formulas with active ingredients (1.8 g protein/100 kcal with prebiotics, probiotics, or both); and was 42%, 49%, and 9% among breastfed infants (Figure 3), respectively.

### 3.5. Formula Intake

**Figure 4:**
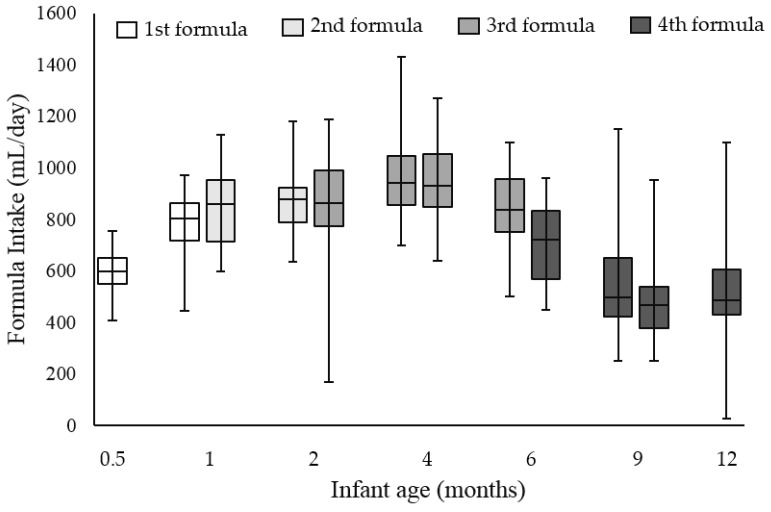
Boxplots of daily intake of study formulas. Data are presented as the average intake per day over three days recorded in the parent-held formula intake booklets. *n* = 29, 28, 27, 29, 28, 28, 27, 28, 25, 28, 26, and 25, respectively.

The median (IQR) daily intake of the 1st study formula increased from 600 (550–652) mL at the age of 0.5 months, to 804 (718–863) mL at the age of one month (Figure 4). The median (IQR) daily intake of the 2nd and 3rd study formulas were relatively stable from month two, through to month four. A modest decrease was observed between four and six months of age, most likely because infants’ diets became more diversified as complementary foods were allowed after the age of four months. The intake decreased substantially to 487 (430–607) mL at the age of 12 months.

### 3.6. Adverse Events

Overall, 164 AEs were reported for 29 (91%) infants (Table S1). Of 164 AEs, only seven mild events, including constipation, hard stools, and flatulence, were reported as probably related to the study formula. Three serious AEs (SAEs), including viral infection, respiratory tract infection, and gastroenteritis rotavirus, were reported for three infants during the study. These SAEs were considered to be unrelated to the study product, as determined by study physicians.

## 4. Discussion

Breast milk composition varies with the duration of lactation [2]. To our knowledge, the innovative nutrition system used in this study represents the first attempt to match multiple aspects of the dynamic nutrition pattern provided in human milk over the course of the first year of life. This study demonstrated that the staged formula system, including an automated formula preparation system and four formulas with an evolving nutrition profile similar to changes in human milk from birth to 12 months of age, (i) is safe and well-tolerated; (ii) supports age-appropriate healthy growth (compared to the WHO growth standards); and (iii) supports an early weight gain pattern from birth to the age of four months, close to infants fed formulas with 1.8 g protein/100 kcal [18], the lowest regulatory-permissible protein level in infant formula in the United States and Europe [23,24,25].

The study demonstrated the safety and tolerability of the study formulas, as indicated by the limited number of mild gastrointestinal AEs, the high formula compliance rate, and low participant dropout rate throughout the study. Overall, only three SAEs were reported in the study, and none of these were related to formula consumption. Moreover, the study demonstrated that the growth in infants exclusively fed the study formulas was non-inferior to the WHO 2006 Growth Standards, at the age of four months. During the entire study period, the upper and lower limits of 95% CIs for all of the anthropometric *z*-scores were within ±1 SD of the WHO growth standards. 

At ages 0.5, 1, and 2 months, the upper limits of the 95% CIs for WAZ and LAZ in the current study were below 0, indicating that infants in the current study who were fed the 1st and 2nd study formulas, had lower mean weight and length values than those included in the WHO Multicentre Growth Reference Study [26]. These findings, however, were acceptable because (i) the cohort of infants was already relatively lighter and shorter than the WHO standards at enrollment (e.g., mean (SD) WAZ and LAZ at enrollment were −0.31 (0.87) and −0.59 (0.98), respectively); (ii) the estimated means and lower limits of the 95% CIs for WAZ and LAZ at age 0.5, 1, and 2 months, were well above −1 SD (−2 SD is the cutoff for malnutrition); and (iii) the estimated mean WLZ and BMIZ at these ages did not differ from 0, therefore tracked closely with the WHO standards. It also appeared that WAZ, LAZ, and BMIAZ decreased from 0.5 to one month of age, by less than 0.5 SD; however, this decline may have been due to low data availability at the age of 0.5 months (~50% of that at other time points). The age-0.5 month visit was close to the enrollment visit (~age 8.7 days); in some cases, these visits were within one to two days of each other, and therefore, the infant did not return for the age-0.5 month visit. 

At four and six months, WAZ, LAZ, WLZ, and BMIAZ were all comparable to the WHO standards. After six months, WAZ, WLZ, and BMIZ gradually increased over time and the lower limits of the 95% CIs were above 0 at nine and 12 months for WAZ and WLZ, and only at 12 months for BMIZ. From four months onwards, infants’ diets became more diversified as complementary foods were allowed in the study. The consumption of complementary foods likely played an increasingly important role in fueling the growth from 6–12 months of life, as daily formula consumption decreased substantially (~50%) in this study. Notably, this observed growth pattern is highly consistent with that reported in the European Childhood Obesity Trial (ECOT) [27], where the means of WAZ, WLZ, and BMIAZ of infants from five European countries (Belgium, Germany, Italy, Poland, and Spain) were initially below the median values of the WHO growth standards, but rose during the second six months of life, to be above the median. This was the pattern for both breast-fed infants in the reference group and formula-fed infants, regardless of the type of formula in the ECOT. The WHO growth standards are based on the data from well-nourished infants from six countries (Brazil, Ghana, India, Norway, Oman, and the USA), who were exclusively breast-fed for the first six months of life. The deviation from the WHO standards observed in Swiss infants in the current study and in the ECOT, may be mainly due to weaning practices. The WHO encourages the initiation of weaning at six months of age; however, the current study permitted weaning at the age of four months, which is a common practice in Europe [28]. 

Accelerated weight gain during infancy has been shown to be a risk factor for obesity in early childhood, adolescence, and even adulthood [17,29]. A foremost postulation for this association has been the “Early Protein Hypothesis”, which suggests that infants fed with a higher protein diet may be at an increased risk of obesity and chronic disease later in life [30]. Protein requirements fall from 1.95–2.04 g/kg body weight/day at the age of one month, to 1.19–1.25 g/kg/day at the age of three months, and 1.05–1.11 g/kg/day at the age of six months [31]. The conventional formulas may provide a more than adequate amount of protein, which is reflected in high plasma amino acids, plasma insulin, and elevated blood urea nitrogen in formula-fed infants, compared with breast-fed infants [32]. Thus, the staged nutrition system evaluated in this study may offer a more physiologic approach (more closely mimicking the dynamic nutritional composition of human milk) for addressing concerns of excess protein consumption in formula-fed infants. We explored the weight gain pattern from birth to four months of age (when infants were still exclusively fed study formulas) in the study. It appeared that infants in the study tended to grow at a slightly faster rate compared to infants exclusively fed formulas of 1.8 g protein/100 kcal with/without other active ingredients, from soon after birth to four months of age, in a recent large-scale pooled analysis [18]. Specifically, a higher proportion of infants in the present study was in the “gradual” weight gain category and a lower proportion of infants was in the “slow” weight gain category. This may be due to the higher protein content in the tested formulas (2.25, 2.0, and 1.88 protein/100 kcal). It is noteworthy that the proportion of infants in the “rapid” weight gain category in the current study was close to that of formula-fed infants in the pooled analysis [18]. The level of 1.8 g protein/100 kcal represents the lowest regulatory allowable limit for protein in infant formula in the United States and the European Union [23,24,25]. This finding suggests that the feeding approach achieved by the staged-formula system may be as effective in impacting “rapid” weight gain pattern in early infancy, as consuming formulas containing 1.8 g protein/100 kcal; however, further studies are needed to test this hypothesis. No data on the WAZ change from birth to 12 months of age were reported in the pooled analysis or, as far as we know, in the existing literature, which restricted our ability to further compare and interpret the results related to the WAZ change between birth and 12 months of age.

The preparation system for the formulas employed in this study was unique. Instead of measuring the powder formula by scoop and reconstitution with added water, this study employed an automated formula preparation system in which a single-serve formula capsule, containing a precise amount of powdered formula, was inserted into the reconstitution system, and appropriate water was automatically added to deliver hygienic reconstituted formula [33]. Since formula preparation by parents has a high probability of either over-concentration or over-dilution [12,13], the current system is much more reliable. The development of the system, coupled with single-serving capsules, also made it more convenient in providing age-appropriate nutrition to infants in real-world settings. 

This is the first infant study testing a nutrition system including an automated formula preparation system and single-serving capsules of formulas, with an evolving nutrition composition which mirrors that in human milk. The innovative system offers an alternative and more physiologic approach of delivering infant nutrition compared with conventional formula feeding. Several limitations warrant a mention. It was not a double-blind, randomized, controlled study. The lack of data on the intake of complementary foods limits our ability to interpret the growth results after four months of age. The enrollment was terminated before the enrollment target had been achieved, mainly due to the high breastfeeding rate of Switzerland. The Swiss Infant Feeding Study [34] reported that 71% of infants were exclusively breastfed in the 1st and 2nd months of life. However, due to the lower than hypothesized data variation (~33%; SD = 0.8 for the WAZ at four months of age obtained in the study vs. hypothesized SD = 1.2 during sample size calculation), the study was still considered adequately powered for testing the primary non-inferiority hypothesis. No biomarkers (e.g., serum amino acids, insulin, and blood urea nitrogen) were assessed in the study. In order to better understand the potential short-term and long-term physiologic benefits of formulas with an evolving nutrition composition, future randomized, double-blind, and controlled studies are needed to compare this new feeding approach with conventional formula-feeding and exclusive breastfeeding.

## 5. Conclusions

To our knowledge, this is the first infant study investigating the growth and safety of infants fed with age-based formulas, with evolving compositions that mirror human milk composition over the course of lactation. Our findings not only show that this stage-based infant formula system is safe and well-tolerated, but also demonstrate that infants fed with the stage-based infant nutrition system grow in agreement with the WHO growth standards and manifest a healthy early weight gain pattern. 

## Figures and Tables

**Table 1:** Composition of study formulas ^1^.

Nutrition Composition	Formulas			
	1 Month	2 Months	3–6 Months	7–12 Months
Energy (kcal/100 mL)	67	65	63	63
Protein (g/100 mL)	1.51	1.31	1.18	1.17
Protein (g/100 kcal)	2.25	2.00	1.88	1.85
Whey:Casein ratio	70:30	70:30	70:30	50:50
Lipids (g/100 mL) ^2^	3.78	3.37	3.22	3.53
Carbohydrates (g/100 mL)	6.74	7.35	7.32	6.62
Lactose (%)	100	100	100	100
Minerals				
Sodium (mg/100 mL)	30.22	27.60	24.48	23.65
Calcium (mg/100 mL)	52.95	46.42	44.06	46.12
Iron (mg/100 mL)	0.72	0.68	0.64	1.02
Zinc (mg/100 mL)	0.76	0.70	0.67	0.57
Vitamins				
A (μg/100 mL)	69.27	67.75	62.42	69.78
D (μg/100 mL)	0.95	0.94	0.90	0.89
E (μg/100 mL)	1.26	1.20	1.28	1.47
B-6 (mg/100 mL)	0.03	0.03	0.03	0.04
B-12 (μg/100 mL)	0.18	0.17	0.18	0.15
Folic acid (μg/100 mL)	11.96	11.92	9.80	10.88

^1^ All values are means or percentage (%); ^2^ The source of lipids was a mixture of milk fat, sunflower oil, rapeseed oil, high-oleic sunflower oil, coconut oil, fish oil (source of docosahexaenoic acid) and fungal oil (source of arachidonic acid).

**Table 2:** Baseline characteristics.

Characteristics	Per Protocol Set (*n* = 30)
Infants, mean (SD) or percentage (%)	
Age at enrollment, days	8.7 (4.2)
Sex, % male	50%
Gestational age, weeks	38.7 (1.0)
Mode of delivery, % Caesarean	57%
APGAR scores at birth, after 5 min	9.1 (1.3)
APGAR scores at birth, after 10 min	9.6 (0.9)
Infant anthropometrics at birth, mean (SD)/median (range)	
Weight-for-age *z*-score	0.04 (0.86)/0 (−1.49, 1.76)
Length-for-age *z*-score	−0.10 (1.04)/−0.08 (−2.23, 1.65)
Weight-for-length *z*-score	0.28 (0.93)/0.17 (−1.71, 2.75)
Body mass index (BMI)-for-age *z*-score	0.16 (0.87)/0 (−1.10, 2.65)
Head circumference-for-age *z*-score	0.54 (0.93)/0.42 (−0.76, 2.64)
Infant anthropometrics at enrollment, mean (SD)/median (range)	
Weight-for-age *z*-score	−0.31 (0.87)/−0.22 (−1.9, 1.57)
Length-for-age *z*-score	−0.59 (0.98)/−0.67 (−2.84, 1.27)
Weight-for-length *z*-score	−0.04 (0.71)/0.02 (−1.86, 1.13)
BMI-for-age *z*-score	−0.03 (0.74)/−0.01 (−1.47, 1.43)
Head circumference-for-age *z*-score	0.18 (0.99)/0.16 (−1.31, 2.04)
Mothers, mean (SD) or percentage (%)	
Age at enrollment, years	35.0 (4.7)
Pre-pregnancy BMI, kg/m^2^	23.30 (2.57)
Mother smoked during pregnancy or after delivery	13%

## Supplementary Materials

The following are available online at http://www.mdpi.com/2072-6643/9/3/219/s1, Table S1: Adverse events.
