# Supplementary material for: Growth of Infants Fed Formula with Evolving Nutrition Composition: A Single-Arm Non-Inferiority Study
Source: Nutrients. 2017 Mar 1;9(3):219. doi: 10.3390/nu9030219 (PMC5372882; doi:10.3390/nu9030219)
Supplement: Supplementary file 1 [file nutrients-09-00219-s001.docx]

Supplementary Materials: Growth of Infants Fed Formula with Evolving Nutrition Composition:
A Single-Arm Non-Inferiority Study

Johannes Spalinger, Andreas Nydegger, Dominique Belli, Raoul I. Furlano, Jian Yan,
Jerome Tanguy, Sophie Pecquet, Frederic Destaillats, Delphine Egli and Philippe Steenhout

**Table S1.** Adverse events ^1, 2^.

| **Adverse Events** | **Full Analysis Set (*n* = 32)** | |
| --- | --- | --- |
|  | **# Events** | **# Infants (%)** |
| All serious and non-serious adverse events in the first 12 months | 164 | 29 (90.6) |
| All serious adverse events in the first 12 months | 3 | 3 (9.4) |
| Select Adverse Events | | |
| Gastrointestinal disorders | 48 | 22 (68.8) |
| Abdominal pain | 2 | 2 6.3) |
| Constipation | 14 | 8 (25.0) |
| Diarrhea | 16 | 10 (31.3) |
| Feces hard | 1 | 1 (3.1) |
| Flatulence | 6 | 3 (9.4) |
| Gastroenteritis | 2 | 1 (3.1) |
| Gastroesophageal reflux disease | 1 | 1 (3.1) |
| Toothache | 3 | 3 (9.4) |
| Vomiting | 3 | 2 (6.3) |
| Infections and infestations | 59 | 21 (65.6) |
| Bronchiolitis | 1 | 1 (3.1) |
| Eye infection | 1 | 1 (3.1) |
| Fungal infection | 1 | 1 (3.1) |
| Gastroenteritis rotavirus | 1 | 1 (3.1) |
| Respiratory tract infection | 46 | 19 (59.4) |
| Rhinitis | 1 | 1 (3.1) |
| Viral infection | 8 | 8 (25.0) |

^1^ Data presented are counts or percentage (%); ^2^ Adverse Events are reported as Preferred Terms under two System Organ Class categories according to the Medical Dictionary for Regulatory Activities (MedDRA) version 4.1.
